# Development of PBS/Nano Composite PHB-Based Multilayer Blown Films with Enhanced Properties for Food Packaging Applications

**DOI:** 10.3390/ma17122894

**Published:** 2024-06-13

**Authors:** Francesco Palmieri, Joseph Nii Ayi Tagoe, Luciano Di Maio

**Affiliations:** 1 Department of Industrial Engineering (DIIN), University of Salerno, Via Giovanni Paolo II, 132, 84084 Fisciano, SA, Italy; frpalmieri@unisa.it; 2 Department of Chemistry and Biology “A. Zambelli”, University of Salerno, Via Giovanni Paolo II, 132, 84084 Fisciano, SA, Italy; jtagoe@unisa.it

**Keywords:** biodegradable polymers, PBS/PHB, multilayer film, nanocomposites, blown films, food packaging, barrier properties

## Abstract

Biobased and biodegradable plastics have emerged as promising alternatives to conventional plastics offering the potential to reduce environmental impacts while promoting sustainability. This study focuses on the production of multilayer blown films with enhanced functional properties suitable for food packaging applications. Films were developed through co-extrusion in a three-layer film configuration, with Polybutylene Succinate (PBS) and Polybutylene Succinate Adipate (PBSA) as the external and internal layers, respectively. The functional layer consisted of Polyhydroxybutyrate (PHB) enhanced with nanoclays Cloisite^®^ 30B at varying weight ratios. Films were also processed by manipulating the extruder screw speed of the functional layer to investigate its impact on the functional properties. Rheology, mechanical strength, and barrier performance were characterised to establish correlations between processing conditions and functional layer blends (Cloisite^®^ 30B/PHB) on the properties of the resultant films. Rheological test results indicated that the system with 5% Cloisite^®^ had the best polymer/nanofiller matrix dispersion. Mechanical and permeability tests showed that by varying the process conditions (the alteration of the thickness of the functionalized layer) resulted in an improvement in mechanical and barrier properties. Furthermore, the addition of the nanofiller resulted in a stiffening of the film with a subsequent decrease in permeability to oxygen and water vapour.

## 1. Introduction

There has been a paradigm shift towards sustainability in recent years, with governments and international bodies adopting measures to reduce carbon footprints and pollution [1]. Currently, the packaging industry mostly utilizes conventional plastics derived from fossil fuels that can accumulate in the environment and contribute to increased greenhouse gas emissions and pollution if not properly managed at their end-of-life [2]. Therefore, the need arises to rethink food packaging solutions to be oriented towards sustainability [3]. One technique to address this issue is the increased interest in the quest for the development of biodegradable plastics with the inherent ability to decompose through natural processes and the use of microorganisms into carbon dioxide, water, and other natural substances [4]. Biobased plastics, on the other hand, can also help the EU achieve climate neutrality by 2050 by reducing reliance on fossil resources and lowering greenhouse gas (GHG) emissions giving industrial stakeholders a sustainable competitive advantage [5]. Although biodegradable polymers remain promising, their use is still quite limited compared to conventional plastics. This is due to higher production costs, and technological limitations in terms of processability and functional performance [6]. Therefore, they need to be enhanced through appropriate strategies. The literature reports several methods for improving the functional properties of films. For instance, several authors have studied the effect of nanoparticle additions to improve mechanical and barrier properties [7,8,9,10]; in other cases, active functions such as the prevention of pathogen proliferation can be obtained through the application specific antimicrobial coatings [11], or antioxidant agents can be incorporated into the polymeric film in order to minimize the degradative effects of oxygen [12,13,14]. In flexible packaging, it is often necessary to modify or even tailor properties for specific applications. In such cases, blending different polymers through melt processing can be an easily affordable solution. Many works focus on this strategy with the aim of obtaining blends with enhanced properties, taking advantage of the complementary characteristics of the neat materials [15,16,17,18,19]. Another approach that has been studied and is currently performed in the flexible packaging industry is the designing and production of multilayer structures [20,21,22,23,24]. The use of different layers of different polymers has the advantage of having different functionalities like mechanical strength, barrier properties, seal ability, and printability assembled in one film. The design of a multilayer film configuration is a complex task, especially in the field of food packaging where layers of different polymers, each of which contributes a specific function to the overall structure, can be assembled with various technologies [25,26]. The multilayer approach can be combined with the use of nanoparticles as well. Nanoparticles, thanks to their extremely small size and high surface area-to-volume ratio, have unique physical and chemical properties that can greatly improve a variety of features when integrated into a polymer matrix. The introduction of nanoparticles into a polymeric film into a multilayer structure has the potentiality to add further functionalities, opening up an array of possibilities to improve packaging film performance. Moreover, due to the increasing interest in biodegradable polymers for packaging, the academic and industrial research aimed to improve the properties of such polymers through the use of approaches like nanoparticle addition or multilayers structure design is also increasing [27,28]. Among biodegradable polymers, Polybutylene succinate (PBS) is a semi-crystalline polymer with mechanical properties similar to those of polyolefins such as polyethylene [29]. It is a ductile polymer with an elongation at break of more than 300% and an elastic modulus in the range of 300–500 MPa, depending on the crystallinity degree, that is easy to process over a wide temperature range [30]. The copolymer poly butylene succinate-co-adipate (PBSA) possesses more ductility and low crystallinity. Poly(hydroxybutyrate) (PHB) is a short-chain-length polyhydroxyalkanoate (PHA) produced by microbial cells subjected to nutrient stress in the presence of excess carbon [31]. Despite being very sustainable, their uses have been limited by their cost, poor mechanical properties due to brittleness, thermal degradation, and difficult processability [32,33]. In efforts to enhance both the processability and functional properties of PHB, researchers are exploring the incorporation of nanoclays into the polymer matrix. A study revealed that nanoclays facilitate not only improved processability but also enhance barrier characteristics by modifying the crystalline morphology [34,35]. Specifically, in a study focusing on PHB nanocomposites containing different types of nanolayered silicates, PHB matrices were successfully reinforced with nanolayered silicates, resulting in improved mechanical and thermal properties, including load-bearing capacity, thermal transition temperatures, and thermal stability demonstrating that the nanocomposites exhibited superior mechanical performance compared to pure PHB [36]. With regard to barrier properties, studies on PHB along with co-polymer poly (3-hydroxybutyrate-hydroxyvalerate) (PHBV) show a reduction in oxygen and water permeabilities as the nanocomposite content increases in the matrix. Such an effect was associated with the enhancement of the tortuosity of the diffusion path [37,38,39]. Given the unexplored potential of multilayer coextrusion involving PBS and PHB, and the use of nanoparticles, this study aims to develop coextruded multilayer biodegradable films tailored for food packaging applications. Specifically, a three-layer system was investigated to leverage the complementary properties of PBS, PBSA, and PHB. The proposed configuration consisted of PBSA and PBS as internal and external layers, respectively, with a functional layer of PHB reinforced using nanoclay Cloisite^®^ 30B at various weight proportions. The multilayer films were then characterized, focusing attention on the evaluation of thermal, barrier, and mechanical properties.

## 2. Materials and Methods

### 2.1. Materials

Pellets of bio-PBS grade FZ91PM (semi-crystalline, Density = 1.26 g/cm^3^, MFR (190 °C, 2.16 kg) = 5 g/10 min, Tm = 115 °C) and bio-PBSA grade FD92PM (semi-crystalline, Density = 1.24 g/cm^3^, MFR (190 °C, 2.16 kg) = 4 g/10 min, T_m_ = 84 °C) were supplied by PTT MCC Biochem (Chatuchak, Bangkok, Thailand). PHB grade Y3000P (specific gravity = 1.25, MFR (190 °C, 2.16 kg) = 10–25 g/10 min, T_m_ = 175–180 °C) was supplied by Tianan-ENMAT™ while Cloisite^®^ 30B (C30B) Nanoclay provided by Southern Clay Products, Inc. (St. Louisville, KY, USA), with a basal interlayer spacing equal to d001  =  18.5 Å and organically modified by methyl, tallow, bis-2-hydroxyethyl quaternary ammonium chloride, was used as nanofiller.

### 2.2. Preparation of the Nanocomposites

The nanocomposite blends were prepared as follows: dry blends of PHB pellets and C30B powder were prepared according to the compositions reported in Table 1; the dry blends were then vacuum-dried at 80 °C overnight. The mixture was compounded by means of a co-rotating Collin ZK25 twin-screw extruder (COLLIN Lab & Pilot Solutions GmbH, Maitenbeth, Germany, D = 25 mm, L/D = 42) at a screw speed of 250 rpm with a thermal profile between 110 °C and 170 °C. The extruded blend filament was pelletized in a SCHEER SGS 25-E4 lab pelletizer (C. f. SCHEER & CIE, Stuttgart, Germany).

### 2.3. Preparation of the Films

Pellets of virgin polymers along with nanocomposite blends were vacuum-dried for 16 h at 80 °C before being film-blown using a laboratory co-extrusion blown film apparatus equipped with three single-screw extruders (Gimac, Castronno, Italy, Dscrew = 12 mm, L = 24 D) and a take-up/cooling system (Collin Film Blowing Line Type BL 50).

**Figure 1:**
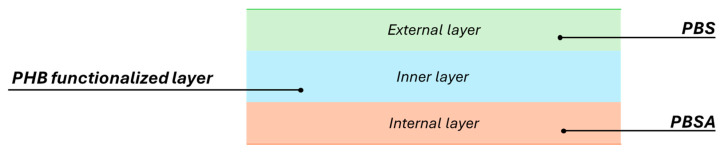
The layout of the produced films.

Three-layer films were produced with a blow-up ratio (BUR) equal to 2.12 with PBS and PBSA as the external and internal layers, respectively, with PHB0, PHB2, and PHB5 sandwiched as the functional layer (Figure 1). The films were processed at constant extruder screw speeds of 50 rpm for the internal and external layers, whereas the screw speed of the extruder for the functional layer ranged from 30 to 60 rpm. The temperature profiles adopted during the blown filming process for each material are shown in Table 2. As reported in Table 3, by increasing the screw speed of the functionalised layers, an increase in the thickness of the films can be achieved., while Table 4 shows images of the samples obtained.

### 2.4. Rheological Characterization

For the observation of the rheological behaviour of the neat and compounded pellets, an Advanced Rheometric Expansion System (ARES) (RHEO Service GmbH & Co. KG, Reichelsheim, Germany) was used to measure the storage modulus G′, the loss modulus G″, and the complex viscosity η*. Before testing, samples were vacuum-dried at 80 °C for 16 h. Tests were performed within parallel plates with diameters of 25 mm and a plate gap of 1 mm under a nitrogen atmosphere. Strain sweep tests were performed between 0.1% and 100% to establish a linear viscoelasticity range followed by a frequency sweep test ranging from 1 to 100 rad/s.

### 2.5. X-ray Diffraction (XRD)

X-ray diffraction patterns were obtained using a Bruker (Milan, Italy) D2 automatic powder diffractometer with nickel-filtered CuKα radiation, operated at a step size of 0.02° with 0.2 s/step.

### 2.6. Differential Scanning Calorimetry

Thermal analysis of the pellets of the pure and compounded materials was performed using a differential scanning calorimeter (Chip-DSC 100, Linseis Messgeraete GmbH, Selb, Germany) under a constant nitrogen flow (100 mL/min). Samples were first heated from 30 °C to 200 °C at a speed of 10 °C/min and held at 200 °C for 5 min, followed by cooling from 200 °C to 30 °C at a speed of 10 °C/min, and second heating from 30 °C to 200 °C at 10 °C/min. The crystallinity degree of the polymers was evaluated as follows:

(1) $$X_{c}=\frac{{∆H}_{m}}{(1-\omega )\times {∆H}_{o}}\times 100\;$$

where ∆H_m_ is the measured heat of fusion, ∆H_o_ is the heat of fusion of a 100% crystalline polymer equal to 195, 146, and 110.3 J/g for PBS, PHB, and PBSA, respectively, and ω is the mass fraction of the nanofiller in the polymer matrix [40,41,42].

### 2.7. Mechanical Properties

Mechanical tensile tests were carried out according to the standard ASTM D882 [43] using a CMT 4000 Series tensile tester (SANS, Macao, China) equipped with a 100 N load cell. Film specimens were cut with a rectangular geometry (width = 12.7 mm and length = 30 mm) along the machine direction. The crosshead speed of the test was kept at 3 mm/min for the duration of each test for the evaluation of the elastic modulus, and at 300 mm/min for the assessment of the percentage of elongation at break.

### 2.8. The Water Vapour Transmission Rate (WVTR)

Water vapour permeability was evaluated using a Systech Illinois water vapour permeation analyser 7002 (Systech Instruments, Thame, UK) according to the ASTM F1249-90 standard [44] at 23 °C and 50% relative humidity. The values of the permeability coefficients were obtained by multiplying the measured value of the WVTR by the respective thickness of the films according to Equation (2).

(2) $$WVP=\frac{WVTR}{P\times \left(R1-R2\right)}\times L\;$$

where L is the average thickness (µm) of the film samples, P is the vapour pressure (bar) of water at 23 °C, and R1 and R2 are the relative humidities (%) of the two chambers.

### 2.9. Oxygen Transmission Rate (OTR)

Oxygen permeability tests were performed using a GTT gas apparatus (Brugger, München, Germany) at 23 °C and 25% R.H., with the oxygen flow rate of 80 mL/min, according to the ASTM D1434 [45] procedure. The sample is placed between two half cells in which a vacuum is created using a pump during the evaluation phase. During the test phase, oxygen gas at a rate of 80 mL/min is passed to the top half cell until it reaches an internal pressure of 1 atm (0.1 Mbar), while the lower half cell stays at a null pressure. In this way, a pressure gradient is created between the two half cells, determining the gas flow through the film to be the gas transmission rate (GTR) with the units (cm^3^/m^2^·d·bar).

## 3. Results and Discussion

### 3.1. Rheology

**Figure 2:**
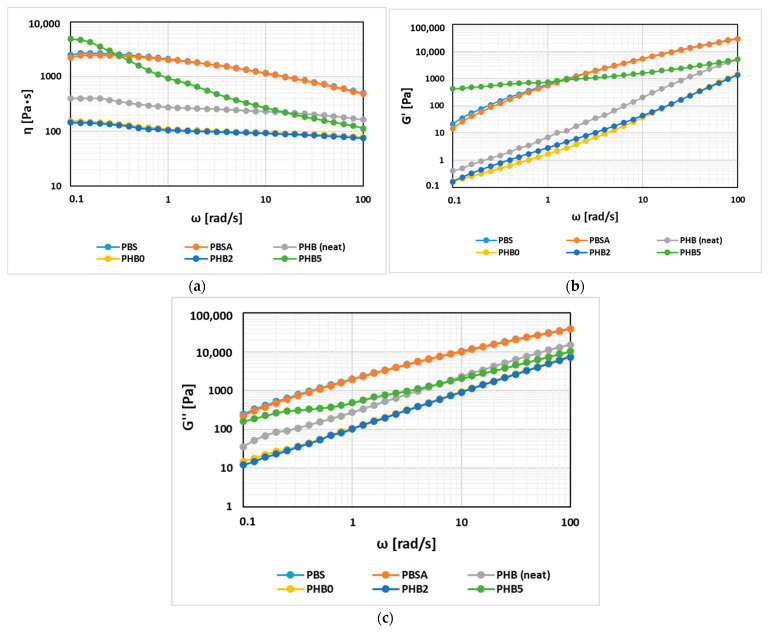
The melt’s rheological response: the complex viscosity (η*) (**a**), storage modulus (G′) (**b**), and loss modulus (G″) (**c**) against the angular frequency (ω).

To investigate the flow behaviour of the polymers for processability in the film-blowing process, a rheological analysis was performed at 180 °C, the results of which are shown in Figure 2.

From the graph illustrating the complex viscosity versus the angular frequency (Figure 2a), the curve for the complex viscosity of PBS, throughout the analysed frequency range, appears to be superimposable on the curve of PBSA, pointing out that despite PBSA being a copolymer with a slightly distinct chemical structure, this may not significantly impact rheological behaviour. The viscosity values of (unprocessed) neat PHB are found to be lower than those for PBS and PBSA. PHB pellets subjected to an extrusion cycle, as seen from the graph (Figure 2a), show a marked decrease in their complex viscosity values; however, the flow curve retains the same trend as that for neat PHB. This could be due to the degradation effect during processing that affects the chain molecular weight and decreasing entanglements. By adding 2% Cloisite, the system shows no noticeable difference, the PHB2 curve turns out to be practically superimposable to that of the non-nano-filled PHB polymer. On the contrary, with the addition of 5% Cloisite, an alteration in the rheological behaviour of the system can be observed. The melt-compounded PHB5 shows a higher complex viscosity than that of PHB0 and PHB2 throughout the analysed frequency range. Additionally, it exhibits a more pronounced shear-thinning behaviour, with a more significant reduction of the Newtonian plateaux than the PHB0 and PHB2 samples. The increased viscosity at higher concentrations of nanoclay proves the development of the intercalated/exfoliated structure of Cloisite 30B which hinders the flow of polymer chains. However, as shear rates increase, the nanoclays can undergo reorientation and can break down temporarily under shearing, hence the convergence while approaching a higher angular frequency. Therefore, incorporating the nanofillers with the PHB matrix led to the enhancement of the processability of the polymers. Similar observations can be made by comparing the behaviour of the storage and loss moduli vs. the angular frequency (Figure 2b,c). Throughout the range of the analysed frequencies, PBS and PBSA show higher G′ and G″ values than the neat PHB. Neat PHB showcases a higher storage (G′) and loss modulus (G″) than the melt-compounded PHB0. As seen for complex viscosity, the values for PHB2 are like those obtained for the processed PHB, while PHB5 shows the most characteristic behaviour; particularly, by observing the trend of G′, a more pronounced solid-like behaviour can be recognized. Generally, when introduced within polymer matrices, nanofillers have a reinforcing effect on properties through the formation of a well-dispersed and oriented nanoclay network. This is evident in this case since, with higher nanoclay contents within the PHB matrix (PHB5), the moduli and viscosity were also increased accordingly, which is consistent with other results in the literature [46].

### 3.2. X-Ray Diffraction (XRD)

**Figure 3:**
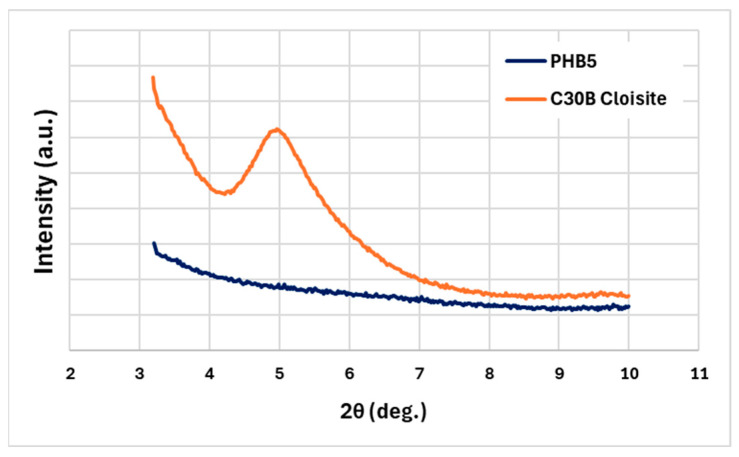
A comparison of the XRD profiles of the Cloisite C30B and the PHB5 pellets.

To verify that Cloisite 30B was dispersed on the nanoscale in the PHB matrix as assumed by the rheological analysis, XRD analysis was carried out on pellets of PHB5. Figure 3 compares the XRD profiles of the organoclay and the PHB5 pellets. As expected, the spectrum of the Cloisite 30B shows a peak centred at about 2θ = 4.96° (d spacing = 17.8 Ǻ). Analysing the XRD profile of PHB5, no peak related to Cloisite can be observed in the explored 2θ range. This agrees with rheological data that suggested the development of the intercalated/exfoliated morphology of the PHB5 system.

### 3.3. DSC Analysis

**Figure 4:**
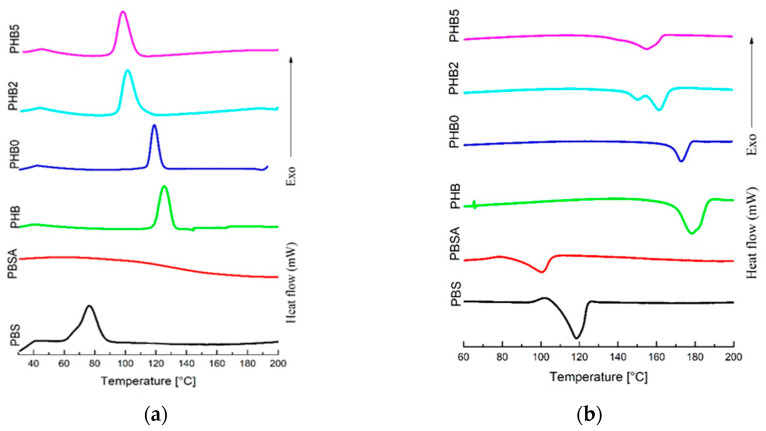
DSC scans for the cooling (**a**) and second heating (**b**) stages of PBS, PBSA, and neat/modified PHB.

The thermal properties of the neat and modified polymers were analysed with DSC as shown in Figure 4. The results summarised in Table 5 show the temperature and enthalpy of crystallisation (T_c_, ∆H_c_), with the melting temperature and melting enthalpy denoted as T_m_ and ∆H_m_, respectively, and the crystallinity degree (X_c_) (calculated considering the heat of fusion of the second heating scan). The crystallinity of PBSA is nearly 50% lower than that of PBS, confirming the existing literature’s assertion that PBSA is more amorphous, contributing to its lower melt temperature [30]. The DSC analysis highlights significant differences in the thermal properties between neat PHB and melt-processed PHB. As reported in Table 5, in subjecting PHB to an extrusion process, the melting temperature decreases from the 178.4 °C of the neat PHB to the 172.9 °C of the PHB0. These behaviours can be easily explained by recalling that the thermomechanical stresses rising during extrusion processes, in general, determine a slight degradation in the polymeric materials. Therefore, a small reduction in molecular weight with consequent changes in thermal transitions is expected [47,48]. By adding Cloisite, a similar behaviour is observed: the melting temperature decreases from the 172.9 °C of PHB0 to the 161.4 °C of PHB2 and the 155.0 °C of PHB5. The addition of C30B also affects the degree of crystallinity of the PHB matrices, X_c_ decreases from the 46.1% of PHB0 to the 42.3% of PHB2 and the 32.3% of PHB5. The decrease in melting temperature and crystalline degree with the increasing nanofiller concentration in PHB shows that the presence of C30B affects polymer chain reorganization, potentially hindering polymer chain mobility, thereby not allowing the reorganization of macromolecules to form crystalline structures. A similar study focusing on the impact of the thermal properties of PHB modified by nanoclay confirms this observation [49].

### 3.4. Mechanical Testing

**Figure 5:**
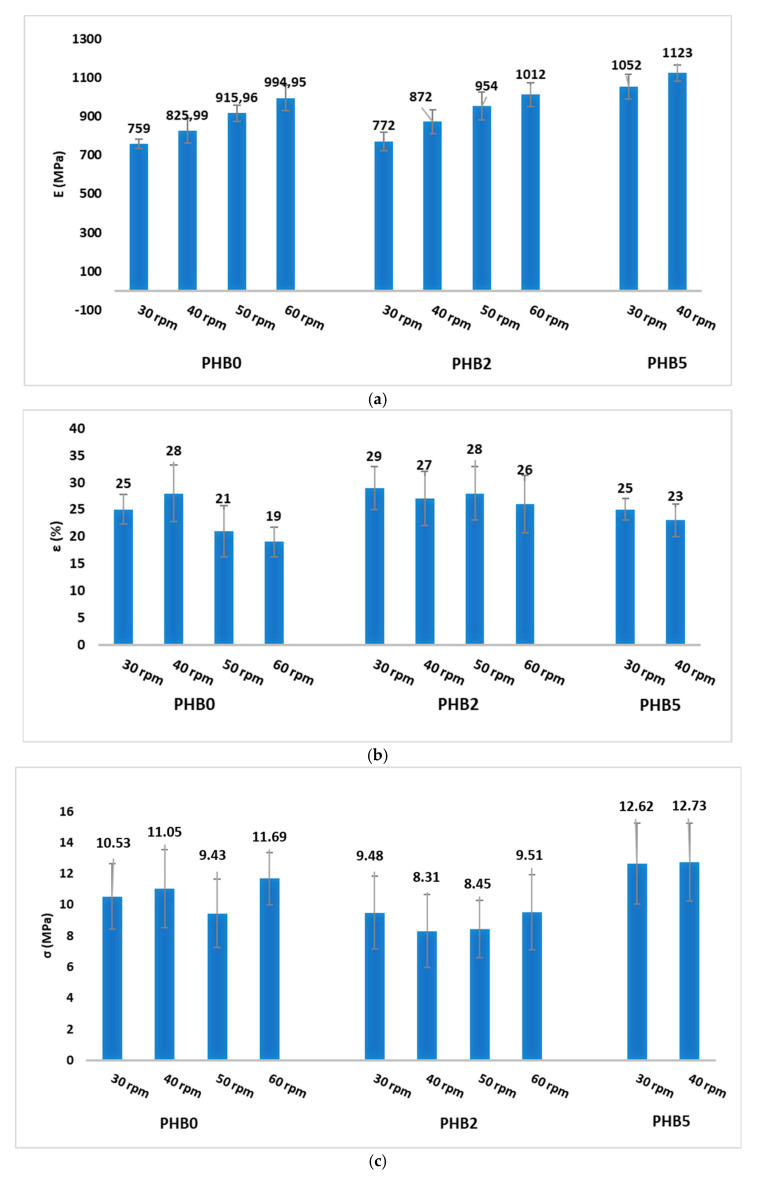
The elastic modulus (**a**), strain at break (**b**) and tensile at break (**c**) of the multilayer films.

Mechanical tests were performed on the neat and multilayer films to determine the elastic modulus and the elongation at break of neat PBS, PHB, and PBSA films as well as the multilayer films produced. The results of the neat films are presented in Table 6, aligning with the anticipated outcomes and the data in the literature [50,51]. Notably, the ductility of PBS and its copolymer PBSA is evident, showcasing an elongation at break exceeding 400%. On the other hand, PHB exhibits a high modulus of elasticity (1456 MPa) and a 4% elongation at break, outlining its characteristic stiffness and brittleness, respectively. However, the properties of the neat films seem to be balanced within the multilayer films as illustrated in Figure 5. Taking into consideration the modulus of elasticity as seen in Figure 5a, there is a general trend in the rise of the Young’s modulus with a significant increase in the film rigidity as the extrusion speed of the functionalized PHB layer increases. The presence of the nanofiller C30B influenced the film properties through a further increase in the elastic modulus, particularly evident in the PHB5 sample. This may be attributable to the development of the intercalated/exfoliated nanocomposite structure characterized by a strong interfacial interaction between the polymers and the nanofillers [52]. This type of structure limits the molecular mobility of PHB macromolecules, leading to a decrease in matrix crystallinity (as discussed in the results of DSC) but, at the same time, to an increase in film stiffness. As expected, the strains at break obtained for the multilayer films are not comparable to those of the neat PBS and PBSA films. However, they remain significantly higher than those obtained for the neat PHB film. As shown in Figure 5b, it is possible to observe that the extrusion speed of the PHB layer does not noticeably affect the strain-at-break values. However, observing the effect of Cloisite, the PHB2 films exhibit a better strain at break than the PHB5 films, probably due to the greater stiffness of the latter film. These results point out that balancing the desired mechanical properties during the integration of additional layers is an important factor in maximizing the performance of these multilayer films. The tensile-at-break values are shown in Figure 5c. By varying the extrusion speed of the functionalized layer (by keeping the amount of Cloisite constant), no great differences in the strain values are visible. The addition of 2% nanoclay does not lead to large differences in the stress values; on the contrary, with 5% Cloisite, a slight increase, attributed to the better dispersion of the nanoclay within the PHB matrix, can be observed.

### 3.5. Oxygen Permeability

**Figure 6:**
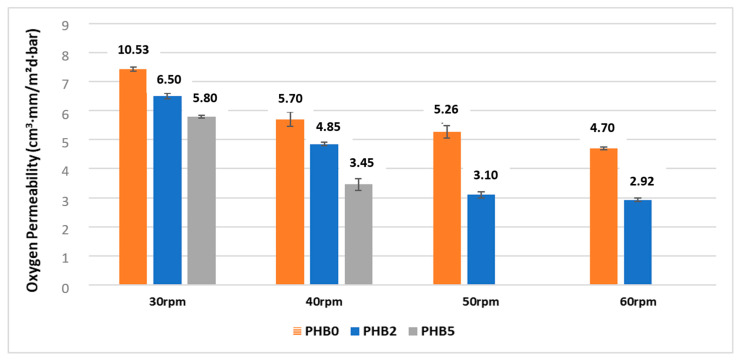
The oxygen permeability of the multilayer films at different screw speeds on the functional layer.

The oxygen permeability of films is an important aspect in evaluating their effectiveness as barriers against oxygen-sensitive foods. The results of the oxygen permeability tests performed on the multilayer films are shown in Figure 6. Table 7 displays the oxygen permeability coefficients of the produced neat films, revealing PBSA, at 36.19 (cm^3^ mm)/(m^2^·d·bar), to have the highest oxygen permeability, which is ten times greater than that of PHB. As is well known, the addition of nanofillers to a polymer matrix leads to improved barrier properties due to their ability to restrict the diffusion of gas molecules by increasing tortuosity [51,52,53,54]. The presented results confirm that an increase in the concentration of C30B nanofiller is associated with a decrease in oxygen permeability. Among the studied samples, the films without the nanofiller presented the highest oxygen permeability values (orange columns). This highlights the influence of the multilayer configuration in the film processing, i.e., for the same percentage of nanoclays, by increasing the thickness of the nanocomposite layer, a progressive decrease in oxygen permeability was observed; for example, the PHB2 multilayer films show values from 6.50 to 2.92 (mm-cm^3^)/(m^2^·d·bar), which corresponds to a percentage decrease of 55%. Similar trends can be observed as the amount of Cloisite content increases. It is worth pointing out that even if, in general, with an increase of film thickness, a decrease of gas transmission rate is normally achieved, in this case, the results show that the permeability coefficient of the multilayer systems also decreases. This is due to the positive effect given by the increase in the overall amount of nanoclay in the films as the thickness of the functionalized layer increases.

### 3.6. Water Vapour Permeability

**Figure 7:**
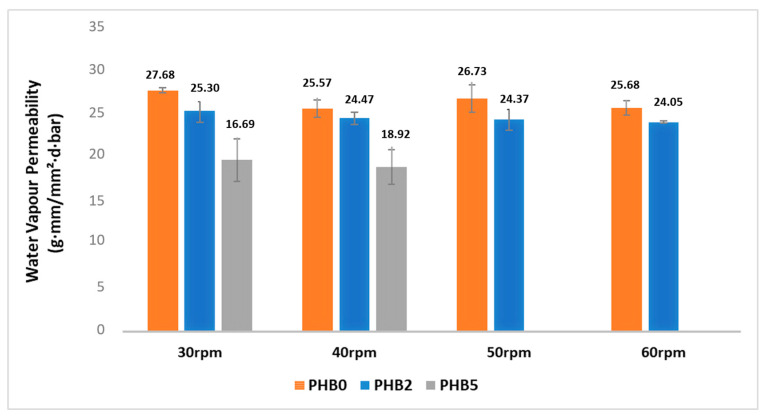
The water vapour permeability based on functional layer variation.

Figure 7 shows the results of the water vapour permeability test on the multilayer films. The first observation is that water permeability is minimally affected by increasing the functional layer thickness at a constant nanofiller loading rate. However, despite the minimal impact of the functional layer’s contents, there is an improvement in barrier performance against water vapour which is clearly attributed to the increased tortuosity and decreased free volume within the film matrix, as also observed in the case of oxygen permeability [37]. The observation of the reduction in water permeability with the increase in the C30B content from 2% to 5% wt underscores the effectiveness of the nanofillers in contributing to a more efficient barrier against water vapour.

## 4. Conclusions

In this work, multilayer blown films suitable for food packaging were successfully produced. The three-layer films were produced with PBS and PBSA as the outer and inner layers, respectively, while the functional layer consisted of a PHB nanocomposite layer containing an organically modified montmorillonite, namely Cloisite C30B. Various mass concentrations of C30B were incorporated into the functional layer, and different extrusion speeds were applied to investigate their impacts on the properties of the resulting films.

The rheological analysis showed that the incorporation of nanofillers C30B and the consequent intercalated/exfoliated morphology generally leads to an increase in viscosity which influences processing conditions. The PHB0 and PHB2 pellets showed comparable trends while PHB5, on the other hand, illustrated a stronger shear-thinning behaviour, which could be associated with a better nanoclay dispersion.

DSC analysis provided insights into the thermal behaviour and crystallinity of these polymers, highlighting the impact of nanoclays and the melt-compounding conditions. The observations revealed reductions in crystallinities and melt peaks with increasing C30B contents, emphasizing the significance of these nanofillers in modifying the thermal properties of polymers.

The mechanical properties (the elastic modulus and the elongation at break) confirmed the influence of both nanoclay contents and the thickness of the functional layer. In particular, the PHB2 films stood out with the best performance, with a 29% elongation at break at 30 rpm. The incorporation of nanofillers and the implementation of the functional layer within a multilayer film exhibits a more complex relationship in tailoring mechanical properties.

Oxygen permeability tests, with a keen focus on neat films and the influences of nanoclay contents in the multilayer films, were performed. The data indicated the role of nanoclays in enhancing the barrier properties of the films against oxygen permeation. The PHB2 film with a functional layer processed at 60 rpm shows the best performance, with an oxygen permeability equal to 2.92 cm^3^ mm/m^2^·d·bar. These findings highlight the potential of optimizing the composition of multilayer films to achieve the desired barrier properties for applications where oxygen transmission needs to be minimized, such as those in food packaging.

The water vapour permeability test demonstrated that although increasing functional layer contents had minimal impact, an improved barrier performance was observed with the increase in C30B mass fractions.

In conclusion, the results show that by increasing the quantity of nanoparticles, although it does not have the best optimal impact on the ductility of films, shows a strong influence on barrier properties. This underscores the potential of utilizing a multilayer configuration involving PBS, PBSA, and nano-filled PHB in food-packaging applications.

## Figures and Tables

**Table 1:** The PHB/Cloisite^®^ 30B blend’s composition.

Mixture	PHB Content [% *w*/*w*]	Cloisite^®^ 30B Content [% *w*/*w*]
PHB0	100	0
PHB2	98	2
PHB5	95	5

**Table 2:** The temperature parameters for extruder zones adopted during the blown filming process.

Mixture	Temperature Profile [°C]			
	T1	T2	T3	T_head_
PBS	150	150	160	*
PHB0	170	170	170	175
PHB2	170	170	165	165
PHB5	170	165	165	165
PBSA	140	140	145	*

* T_head_ is not reported for PBS and PBSA since it is the same as that used for the PHB layer.

**Table 3:** The extruded multilayer films with their relative extruder speeds and thicknesses.

Film Sample	Functional Layer	Functional Layer Extruder Speed (rpm)	Film Thickness (mm)
1	PHB0	30	0.059 ± 1
2		40	0.062 ± 1
3		50	0.066 ± 1
4		60	0.072 ± 1
5	PHB2	30	0.060 ± 1
6		40	0.063 ± 1
7		50	0.068 ± 1
8		60	0.075 ± 2
9	PHB5	30	0.060 ± 1
10		40	0.064 ± 1
11		*	*
12		*	*

* Films appeared grainy with poor bubble stability during extrusion, making samples difficult to collect.

**Table 4:** Images of the films produced.

Functional Layer	30 rpm	40 rpm	50 rpm	60 rpm
PHB + 0% C30B	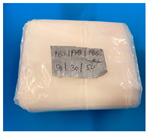	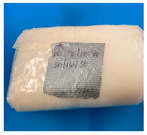	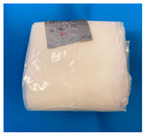	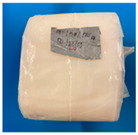
PHB + 2% C30B	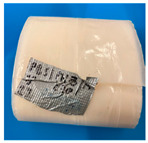	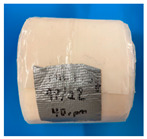	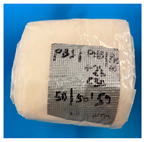	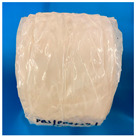
PHB + 5% C30B	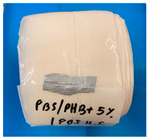	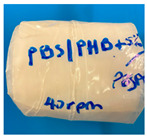	-	-

**Table 5:** The thermal properties of the pellets.

Sample	T_c_ [°C]	ΔH_c_ [J/g]	T_m_ [°C]	ΔH_m_ [J/g]	X_c_ [%]
PBS	75.5	46.6	118.5	69.7	35.7
PBSA	-	-	100.6	23.7	21.5
PHB (neat)	125.5	70.0	178.4	73.0	50.0
PHB0 (melt-processed)	119.0	69.3	172.9	67.3	46.1
PHB2	101.5	55.7	161.4	61.7	42.3
PHB5	98.4	49.8	155.0	47.2	32.3

**Table 6:** The mechanical properties of the neat films *.

Sample	Modulus of Elasticity [MPa]	Strain at Break [%]
PBS	504 ± 22	409 ± 24
PHB	1456 ± 79	4 ± 1
PBSA	215 ± 16	553 ± 34

* Data were obtained by testing monolayer films produced using the temperature profiles shown in Table 2, with an extrusion speed of 50 rpm for PBS and PBSA, and 30 rpm for PHB.

**Table 7:** The oxygen permeability coefficients of the neat films *.

Mixture	PO_2_ [cm^3^ mm/m^2^·d·bar]
PBS	7.72
PBSA	36.19
PHB	3.25

* Data were obtained by testing monolayer films produced using the temperature profiles shown in Table 2, with an extrusion speed of 50 rpm for PBS and PBSA, and 30 rpm for PHB.

## Data Availability

The original contributions presented in the study are included in the article, further inquiries can be directed to the corresponding author.
